# The Role of Internal Limiting Membrane as a Biomarker in the Evolution of Myopic Traction Maculopathy

**DOI:** 10.3389/fmed.2021.802626

**Published:** 2022-01-07

**Authors:** Dong Fang, Jindi Su, Lu Chen, Shaochong Zhang

**Affiliations:** ^1^Shenzhen Eye Hospital, Jinan University, Shenzhen Key Laboratory of Ophthalmology, Shenzhen, China; ^2^Affiliated Shenzhen Maternity and Child Healthcare Hospital, Southern Medical University, Shenzhen, China

**Keywords:** internal limiting membrane, optical coherence tomography, evolution, biomarker, myopic traction maculopathy (MTM)

## Abstract

**Purpose:** To describe the longitudinal structural changes of myopic traction maculopathy (MTM) based on optical coherence tomography (OCT) and to detect biomarkers in the evolution of MTM.

**Methods:** A retrospective study was conducted on patients with MTM as defined by OCT. A minimum follow-up of 6 months was necessary for study inclusion. The effects of comprehensive OCT-based structure on the evolution of MTM, the progression rates, and resolution rates of MTM were evaluated.

**Results:** A total of 120 eyes (120 patients) were included with an average follow-up of 15.4 months. During the follow-up, MTM progressed in 32 eyes (26.67%). The most common pattern of progression observed was the increased extent of retinoschisis in 12 eyes. The multivariate analysis showed that the presence of MTM progression had a significant correlation with internal limiting membrane (ILM) detachment and retinoschisis involved the entire macula at baseline. Five eyes (4.17%) experienced MTM resolution, of which 2 eyes developed disruptions of detached ILM, two eyes developed disruptions of epiretinal membrane, and one eye developed partial posterior vitreous detachment. Eyes with foveal detachment showed the highest progression rate (41.67%) and highest resolution rate (16.67%) compared to the eyes with other foveal complications.

**Conclusion:** ILM detachment is a risk factor for MTM progression and MTM resolution can occur after ILM disruption. These suggest that ILM may play an important role as a biomarker in the evolution of MTM.

## Introduction

Myopia is a major global public health problem. Approximately, one in six of population of the world is myopic (1). It is expected that nearly half of population of the world may be myopic by 2050, with as much as 10% highly myopic (2). Complications related to high myopia are now a major cause of visual impairment and blindness worldwide, especially in east Asia (3). Among them, myopic traction maculopathy (MTM) is one of the main causes for the progressive vision impairment in patients with high myopia, affecting as many as one-third of eyes with posterior staphyloma (4, 5).

Myopic traction maculopathy was proposed by Panozzo et al. (6) in 2004. It is an umbrella term that encompasses a wide spectrum of pathologic features generated by the traction in the pathologic myopic environment. In addition to the typical myopic tractional changes of myopic retinoschisis, generalized MTM also includes vitreoretinal interface abnormalities and foveal complications generated by traction. The detailed pathogenesis of MTM has not been clarified. However, tractional forces from vitreoretinal interfaces such as vitreomacular traction (VMT), epiretinal membrane (ERM), internal limiting membrane (ILM), or a combination of these are thought to be important factors (7, 8). To study the natural course of MTM, several study groups have conducted follow-up studies on MTM (9, 10). However, long-term follow-up studies on large samples are scarce and the long-term outcome and the biomarkers in MTM evolution remain unclear.

Myopic traction maculopathy covers a range of conditions whose prognoses vary significantly. Previous studies have verified that its prognosis is related to the retinoschisis grading (11). However, optical coherence tomography (OCT)-based structural changes including vitreoretinal interface abnormalities and various types of foveal complications are closely associated with the outcome of MTM. These structural changes were rarely considered in previous studies, thereby leading to an incomplete understanding of the natural history and prognosis of the disease.

In this study, the longitudinal changes of MTM in a large sample of highly myopic Chinese individuals were evaluated. Further, OCT-based structural changes, including the grading of retinoschisis, vitreoretinal interface abnormalities, and foveal complications, were comprehensively analyzed to identify the biomarkers in MTM evolution.

## Methods

This study was approved by the Institutional Review Board of Shenzhen Eye Hospital (Shenzhen China) and was conducted in accordance with the World Medical Association Declaration of Helsinki. The medical records of consecutive patients with high myopia at the Shenzhen Eye Hospital between September 2017 and October 2020 were reviewed. Inclusion criteria were: (1) highly myopic eyes, defined by refractive error [expressed as spherical equivalent (SE)] < −6 D or by an axial length ≥ 26.5 mm; (2) undergone spectral-domain OCT examinations including vertical, horizontal, and radial scans through the fovea; (3) OCT features corresponding to the diagnosis standards of MTM proposed by Panozzo et al. (6); and (4) a minimum follow-up period of 6 months, unless there was either resolution or progression. Exclusion criteria were: (1) eyes with previous vitreoretinal surgery; (2) eyes with any associated or concomitant retinopathy that could confound the retinal interpretation of OCT images; (3) eyes with macular chorioretinal atrophy or myopic choroidal neovascularization; and (4) OCT images with poor quality, which was defined as insufficient visualization of the retinal pigment epithelium (RPE) line in the macular area. Only one eye of each participant was used for analysis. When both eyes of the same individuals were included, the eye with worse best corrected visual acuity (BCVA) was considered for the analysis.

All the patients underwent a comprehensive ophthalmological examination including BCVA, refractometry using autorefractor (KR-8800, Topcon, Tokyo, Japan), fundus photographs (Visucam, Carl Zeiss Meditec, Jena, Germany, UK), and spectral-domain OCT. The axial length of each participant was measured using IOLMaster (Carl Zeiss Meditec, Jena, Germany, UK). OCT imaging was performed with either OCT (Optovue Incorporation, Fremont, California, USA) or Spectralis OCT (Heidelberg Engineering, Heidelberg, Germany, UK). Vertical and horizontal scans, passing through the center of the fovea and radial scans, covering all the macular complications, were performed for each patient in OCT examination. Patients underwent a series of OCT scans spanning in a period of more than 6 months, unless there was either resolution or progression during the follow-up period. All the OCT images were read by an experienced OCT grader (SZ) masked to all the clinical data at the time of grading.

**Figure 1:**
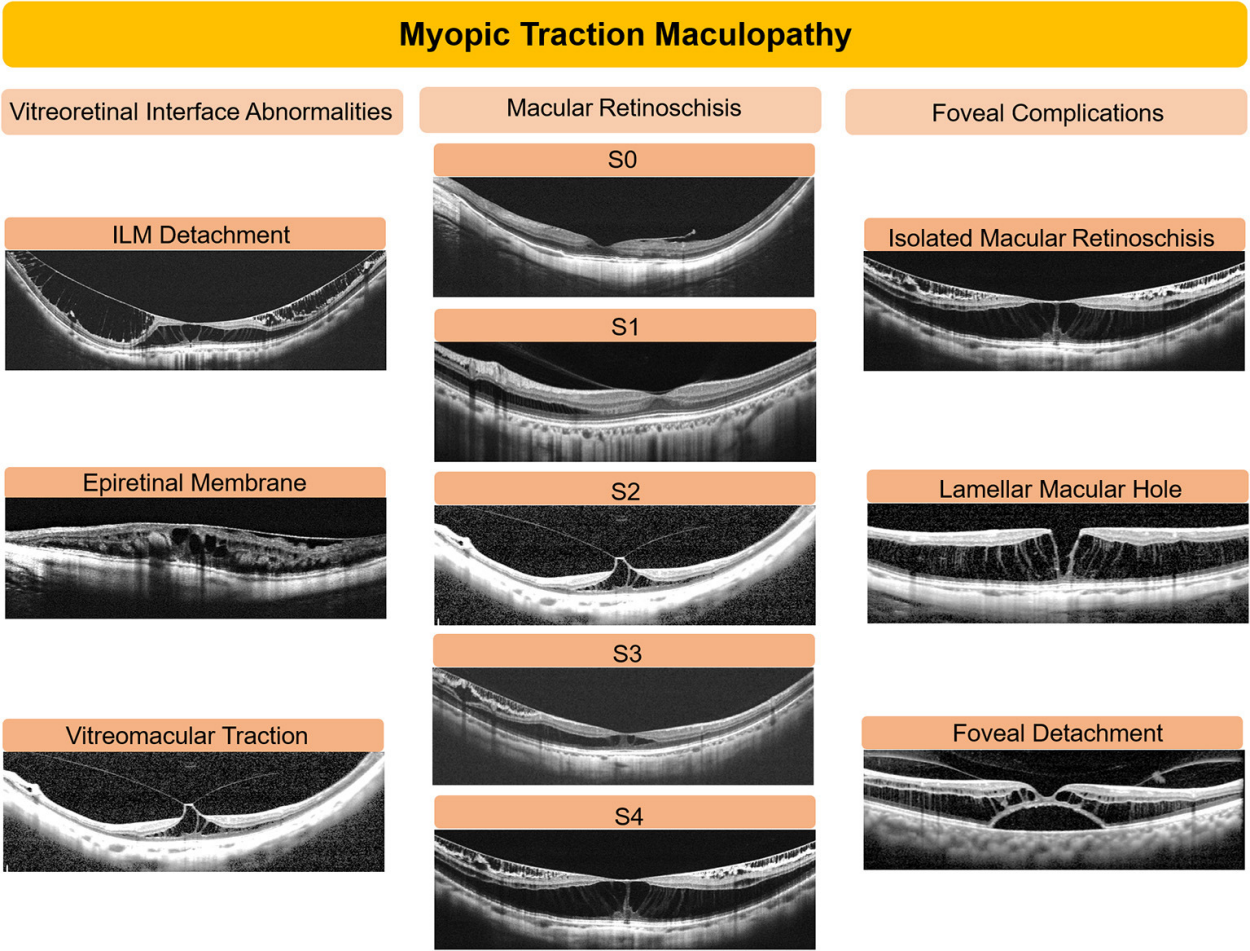
Representative images of optical coherence tomography (OCT) in eyes with myopic traction maculopathy. OCT-based structural changes were comprehensively analyzed based on the vitreoretinal interface abnormalities, degree of retinoschisis, and foveal complications. The outer retinoschisis was graded into 5 levels: no macular retinoschisis (S0), extrafoveal macular retinoschisis (S1), fovea-only macular retinoschisis (S2), foveal but not the entire macular area macular retinoschisis (S3), and entire macular area retinoschisis (S4). Foveal complications included isolated macular retinoschisis without further complications, lamellar macular hole, and foveal detachment. Vitreomacular interface abnormalities included epiretinal membrane, vitreomacular traction, and internal limiting membrane (ILM) detachment.

Changes of OCT-based structure were evaluated in three aspects including retinoschisis grading, foveal complications, and vitreomacular interface abnormalities. Representative images are shown in Figure 1. The outer retinoschisis was graded according to the location and the size as suggested by Parolini et al. (11): no macular retinoschisis (S0), extrafoveal macular retinoschisis (S1), fovea-only macular retinoschisis (S2), foveal but not the entire macular area macular retinoschisis (S3), and entire macular area macular retinoschisis (S4). Foveal complications including lamellar macular hole (MH), foveal detachment, and full-thickness MH were recorded. Vitreomacular interface abnormalities including ERM, VMT, and ILM detachment were also analyzed. MTM was defined as progressed in the event of: (1) for the macular retinoschisis, an increase of more than 100 μm in height or an enlargement of the macular retinoschisis toward the area that did not have a retinoschisis (11); (2) development of any foveal complications including lamellar MH, foveal detachment, and full-thickness MH; and (3) for foveal complications, an increase of more than 100 μm in height or an enlargement toward the area that did not have the lesion at baseline. A resolution of MTM was defined in the event of: (1) a decrease in the height or extent of the macular retinoschisis without any sign of progression and (2) a decrease in the height or extent of foveal complications without any sign of progression. Alternatively, an eye without progression or resolution was defined as stable. The rate of progression and resolution in MTM and the prognostic factors for progression were analyzed.

The BCVA was presented as the logarithm of the minimum angle of resolution (logMAR). Continuous variables between groups were compared using the one-way ANOVA test or the Mann–Whitney *U*-test and the Kruskal–Wallis test. Multiple comparisons between different groups were adjusted by the Bonferroni test. Categorical data were compared using the Pearson's chi-squared test or the Fisher's exact test. The multivariate binary regression analysis was performed with the presence of MTM progression as the dependent variable and age, axial length, the categories of retinoschisis, the presence of foveal complications, the presence of ILM detachment, and tractional lesions as independent variables. *p* < 0.05 was considered as statistically significant. All the data were analyzed using the SPSS 19.0 statistical software (SPSS Incorporation, Chicago, Illinois, USA).

## Results

### Clinical Characteristics of Eyes With MTM at Presentation

A total of 120 eyes of 120 consecutive patients with a diagnosis of MTM were studied. The objects comprised 73 women and 47 men with a mean age of 52.78 ± 9.91 years ranging from 21.0 to 76.0 years. The mean baseline BCVA was 0.42 ± 0.40 with a range of 0 to 1.85. The mean refractive error was −14.27 ± 5.75 D ranging from−5.5 to −29.0 D. The mean axial length was 30.18 ± 2.43 mm with a range of 24.85–35.93 mm.

The baseline characteristics of MTM in each group are shown in Table 1. Among the 120 eyes with MTM, 31 eyes (25.83%), 30 eyes (25.00%), 12 eyes (10.00%), 22 eyes (18.33%), and 25 eyes (20.83%) had grades of S0, S1, S2, S3, and S4, respectively. The 31 eyes with S0 included 22 eyes with an isolated ERM, 4 eyes with an isolated VMT, 2 eyes with an ERM and coexisting VMT, and 3 eyes with subfoveal retinal thickness > 200 mm, but without evidence of a macular retinoschisis. Among the 12 eyes with S2, 9 eyes were accompanied by lamellar MH and one eye was accompanied by foveal detachment. Among the 22 eyes with S3, 8 eyes had coexisting lamellar MH and 3 eyes exhibited foveal detachment. In 25 eyes with S4, 8 eyes presented foveal detachment and 10 eyes displayed lamellar MH, 2 of which showed both the complications. The differences were not significant in age (*p* = 0.05, ANOVA test) and in axial length (*p* = 0.06, ANOVA test) at baseline between the eyes with different categories of retinoschisis (S0–S4). There were significant differences in BCVA among the eyes with different categories of retinoschisis (*p* < 0.001, Kruskal–Wallis test). Patients with S3 and S4 as compared with individuals with S0 and S1 had a significantly worse BCVA at baseline (*p* = 0.006, *p* < 0.001, *p* = 0.03, *p* < 0.001).

**Table 1:** Baseline characteristics of patients with different categories of retinoschisis.

**Characteristics**	**S0 (31 eyes)**	**S1 (30 eyes)**	**S2 (12 eyes)**	**S3 (22 eyes)**	**S4 (25 eyes)**
**Age (y)**					
Mean ± SD	54.35 ± 8.13	50.10 ± 11.13	57.58 ± 6.33	49.45 ± 6.46	54.64 ± 12.72
Range	41–76	26–70	47–71	37–66	21–74
**BCVA (logMAR)**					
Mean ± SD	0.21 ± 0.17	0.25 ± 0.20	0.44 ± 0.29	0.54 ± 0.51	0.78 ± 0.44
Range	0.00–0.70	0.00–0.70	0.10–1.00	0.00–1.85	0.05–1.85
**Axial length (mm)**					
Mean ± SD	29.41 ± 2.61	31.08 ± 2.33	31.09 ± 2.20	29.98 ± 2.12	29.82 ± 2.35
Range	24.85–35.93	25.42–35.47	27.69–34.32	27.03–32.86	26.66–33.48
Progressed (eyes)	2 (6.45%)	5 (16.67%)	3 (25.00%)	8 (36.36%)	14 (56.00%)
Pathology enlargement^*^	0	4	3	4	9
Enlargement of retinoschisis	0	4	2	3	3
Enlargement of LMH	0	0	1	0	2
Enlargement of foveal RD	0	0	0	1	4
Newly onset pathology	3	2	0	4	5
Development of retinoschisis	2	0	0	0	0
Development of LMH	1	2	0	1	1
Development of foveal RD	0	0	0	2	4
Development of FTMH	0	0	0	1	0
Duration of progression	15.5	19.4	15	12.13	11.46
Improved (eyes)	0 (0%)	1 (3.33%)	0 (0%)	3 (13.64%)	1 (4.00%)
Stable (eyes)	29 (93.55%)	24 (80.00%)	9 (75.00%)	11 (50.00%)	10 (40.00%)

*BCVA, best corrected visual acuity; logMAR, logarithm of the minimum angle of resolution; RD, retinal detachment; LMH, lamellar macular hole; FTMH, full-thickness MH*.

* *Increased height or extent of the pathology*.

In terms of foveal complications, eyes were divided into 3 groups including isolated retinoschisis, lamellar MH, and foveal detachment (Table 2). None of the eyes with full-thickness MH was enrolled for their short duration of follow-up. A total of 83 eyes showed isolated retinoschisis, 27 eyes presented retinoschisis and coexisting lamellar MH, and 12 eyes displayed retinoschisis accompanied with foveal detachment (2 eyes had both the lamellar MH and foveal detachment). Similarly, the differences were not significant in age (*p* = 0.71, ANOVA test) and in axial length (*p* = 0.10, ANOVA test) at the initial visit between the eyes with different foveal complications. There were significant differences in BCVA among the 3 groups (*p* < 0.001, Kruskal–Wallis test). Patients with foveal detachment presented a significantly worse BCVA at baseline as compared with individuals with lamellar MH and isolated retinoschisis (*p* < 0.001, *p* < 0.001).

**Table 2:** Baseline characteristics of patients with different foveal complications.

**Characteristics**	**Retinoschisis (83 eyes)**	**LMH (27 eyes)**	**Foveal RD (12 eyes)**
**Age (y)**			
Mean ± SD	52.43 ± 9.54	53.07 ± 11.48	54.92 ± 8.22
Range	26–76	21–72	39–70
**BCVA (logMAR)**			
Mean ± SD	0.30 ± 0.29	0.64 ± 0.39	0.91 ± 0.53
Range	0.00–1.85	0.10–1.30	0.22–1.85
**Axial length (mm)**			
Mean ± SD	29.88 ± 2.52	30.83 ± 2.25	31.11 ± 1.61
Range	24.85–35.93	26.69–34.32	28.85–33.48
Progressed (eyes)	19 (22.89%)	9 (33.33%)	5 (41.67%)
Pathology enlargement^*^	9	7	5
Enlargement of retinoschisis	9	3	0
Enlargement of LMH	0	3	0
Enlargement of foveal RD	0	1	5
Newly onset pathology	12	2	0
Development of retinoschisis	2	0	0
Development of LMH	5	0	0
Development of foveal RD	4	2	0
Development of FTMH	1	0	0
Duration of progression	16.1 ± 3.9	12.3 ± 4.1	2.9 ± 2.3
Improved (eyes)	3 (3.61%)	0 (0%)	2 (16.67%)
Stable (eyes)	61 (73.49%)	18 (66.67%)	5 (41.67%)

*BCVA, best corrected visual acuity; logMAR, logarithm of the minimum angle of resolution; RD, retinal detachment; LMH, lamellar macular hole; FTMH, full-thickness MH*.

* *Increased height or extent of the pathology*.

Vitreomacular interface abnormalities including ILM detachment, ERM, and VMT were evaluated. An ILM detachment was present in 32 eyes (26.67%) and all of them showed concomitant outer retinoschisis. Tractional lesions including ERM and VMT were present in 88 eyes, of which 59 eyes showed isolated ERM, 22 eyes showed isolated VMT, and the remaining 7 eyes showed both the lesions.

### Characteristics in MTM Progression in High Myopia

**Figure 2:**
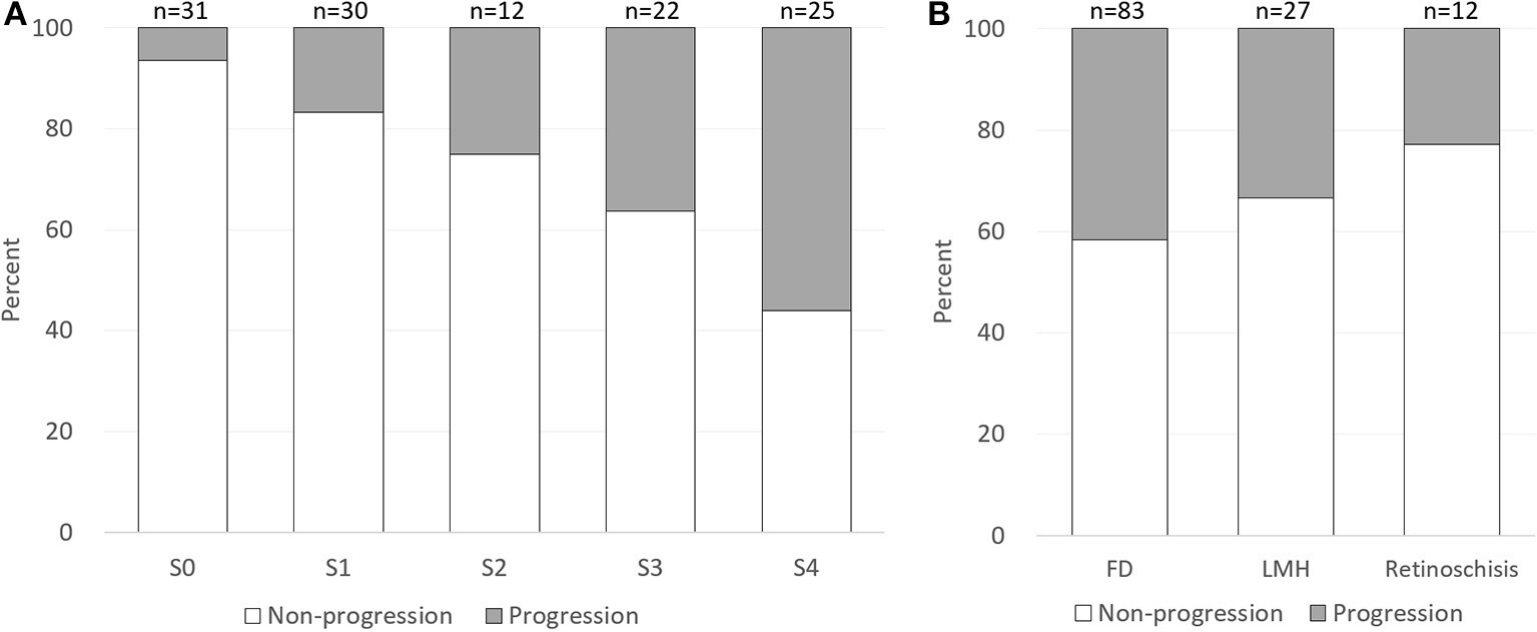
Proportion of myopic tractional maculopathy progression by category of retinoschisis and foveal complications at baseline. **(A)** The progression rate increased with the increasing severity of retinoschisis (S0–S4). **(B)** Eyes with foveal detachment showed the highest progression rate (41.67%), followed by the eyes with lamellar macular hole (33.33%) and the eyes with isolated retinoschisis (22.89%). FD, foveal detachment; LMH, lamellar macular hole.

The mean length of follow-up was 15.4 ± 12.2 months (range, 6–61 months). Among the 120 eyes included, 32 eyes (26.67%) showed MTM progression. Overall, the progression rate increased with the increasing severity of retinoschisis (S0–S4, *p* < 0.001, Fisher's exact test). A summary of the progression of MTM is given in Figure 2A, Table 1. A significantly higher progression rate was found in eyes with S4 as compared with eyes with S0 and S1 (*p* < 0.001; *p* = 0.003).

A total of 14 newly onset lesions developed in 13 eyes in the OCT findings during the follow-up period, including one eye changed from S0 to extrafoveal retinoschisis (S1), one eye changed from S0 to extrafoveal retinoschisis (S1) with coexisting lamellar MH, 4 eyes developed lamellar MH, 6 eyes developed foveal detachment, and one eye developed full-thickness MH. In addition, a total of 20 lesion changes were identified in 20 eyes with progressive MTM. The most common pattern of progression observed was increasing height or extent of macular retinoschisis in 12 eyes, accounting for 60% of the total number of lesions changes and 37.5% of MTM progressions, followed by the enlargement of foveal detachment in 5 eyes (15.63%).

**Figure 3:**
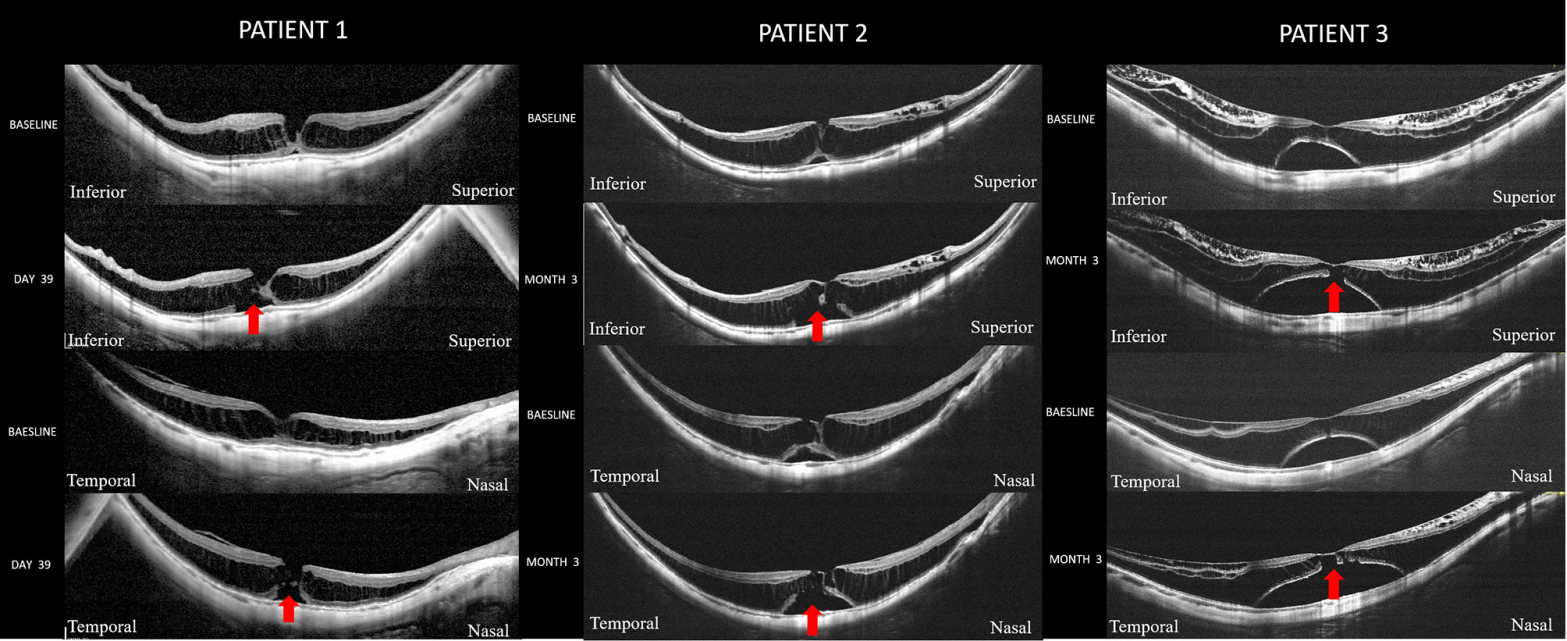
Cases with foveal detachment experienced rapid progression. **Patient 1** is a woman (55-year-old) with a spherical equivalent refractive error of −23.0 D and an axial length of 32.4 mm. Optical coherence tomography (OCT) scan across the fovea at the initial visit showed a retinoschisis spanning the entire macula with a lamellar MH. A slight foveal elevation can be seen. Thirty-nine days later, the extent of foveal detachment has increased and disruption of the outer retina (red arrow) has developed. The best corrected visual acuity (BCVA) declined from the logarithm of the minimum angle of resolution (logMAR) 0.70 to logMAR 1.0. **Patient 2** is a woman (61-year-old) with an axial length of 31.74 mm. At baseline, she had S4 retinoschisis involving the entire macula and a shallow foveal detachment. At the third month of the follow-up, the extent of foveal detachment increased with a disruption of the outer retina (red arrow) developed. The BCVA declined from logMAR 1.00 to logMAR 1.3. **Patient 3** is a woman (50-year-old) with an axial length of 30.21 mm. OCT scan at baseline showed an outer retinoschisis spanning the entire macula with commitment inner retinoschisis and internal limiting membrane detachment. A foveal detachment was observed. 3 months later, the foveal detachment has further enlarged and disruption of the outer retina (red arrow) was noted. The BCVA declined from logMAR 1.00 to logMAR 1.3.

On the other hand, eyes with different foveal complications exhibited different progression rates (Figure 2B, Table 2). Eyes with foveal detachment showed the highest progression rate (41.67%), followed by the eyes with lamellar MH (33.33%) and the eyes with isolated retinoschisis (22.89%). Notably, among the 12 eyes with foveal detachment, 5 eyes experienced progression in a short average duration of 2.9 months (range, 39 days to 7 months). Representative cases of foveal detachment progression are shown in Figure 3. Among them, the eye with the most rapid progression showed an elevation of foveal detachment and a break of outer retina in only 39 days (Figure 3, Patient 1).

In 32 eyes with ILM detachment, progressions of MTM were observed in 13 eyes (40.63%), whereas a lower progression rate (21 eyes, 23.86%) was detected in the eyes without ILM detachment. The eyes with tractional lesions displayed a similar rate of progression compared to the eyes without tractional lesions (29.55 vs. 25%).

### Visual Outcome and Risk Factors for MTM Progression

Mean BCVA of the whole cohort at the end of follow-up was 0.50 ± 0.47, which was comparable to that at baseline (0.42 ± 0.40) (*p* = 0.49, Mann–Whitney *U*-test). For the patients whose MTM had progressed, the BCVA at the final examination (0.81 ± 0.47) was significantly worse than that at the initial examination (0.58 ± 0.45) (*P* = 0.04, Mann–Whitney *U*-test). The BCVA at the initial and final examinations was not statistically different in either the group with stable MTM or the group with resolved MTM (*p* = 0.76, *p* = 0.78, Mann–Whitney *U*-test). The multivariate binary analysis adjusted for age and axial length was done to reveal the risk factors for MTM progression. The results showed that MTM progression had significant correlation with the presence of ILM detachment (*p* = 0.02; odds ratio, 3.68; 95% CI, 1.25–10.84, multivariate binary regression analysis) and eyes with S4 category of MTM at baseline (*p* = 0.004; odds ratio, 3.85; 95% CI, 1.54–9.62, multivariate binary regression analysis).

### Characteristics in MTM Resolution in High Myopia

**Figure 4:**
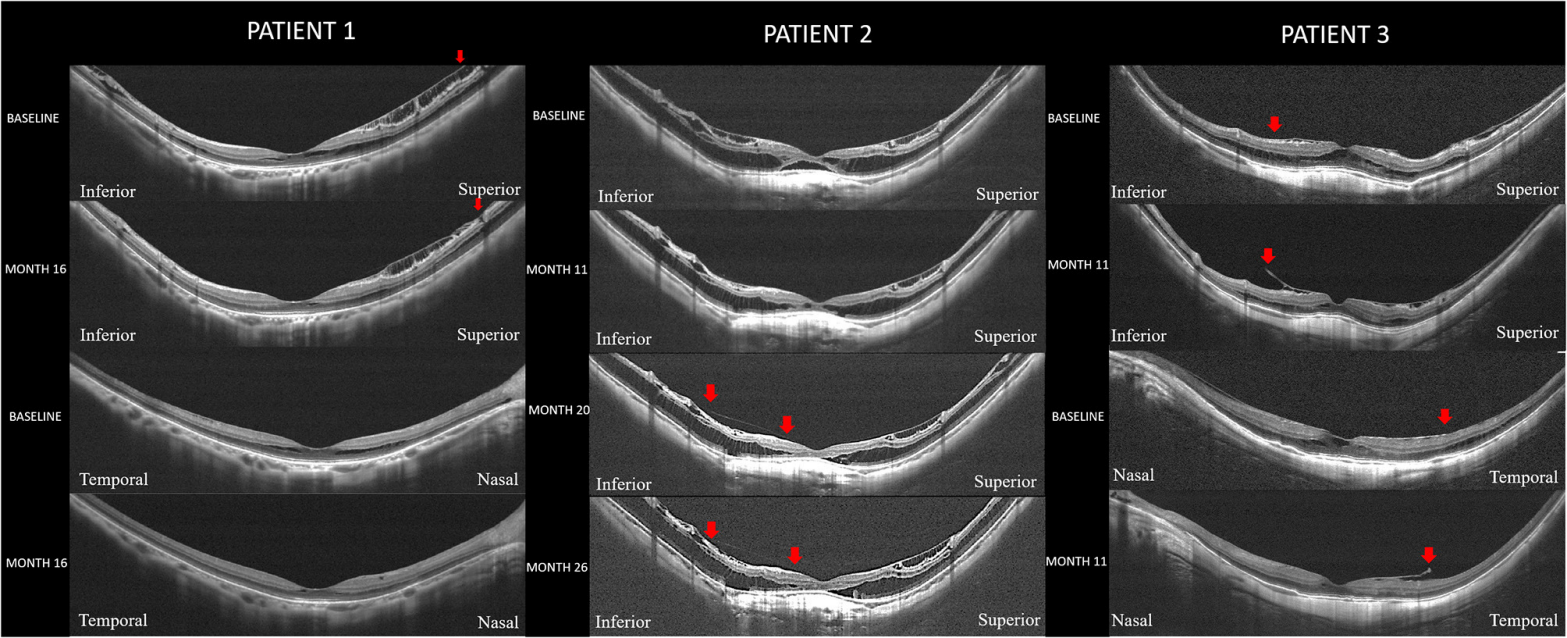
Representative cases with spontaneous resolution of myopic traction maculopathy. **Patient 1:** Resolution of a macular retinoschisis in an eye with a disruption of the internal limiting membrane (ILM). Right eye with an axial length of 27.05 mm in a woman (50-year-old). Optical coherence tomography (OCT) scan in both the vertical and horizontal section at baseline showed a shallow foveal retinoschisis with ILM detachment. Sixteen months later, a disruption of the detached ILM superior to the fovea is observed (red arrow) and the foveal retinoschisis is disappeared. **Patient 2:** Resolution of a foveal detachment in an eye with a partial posterior vitreous detachment (PVD). Right eye with an axial length of 32.86 mm in a man (53-year-old). OCT scan in vertical section at baseline showed a shallow foveal detachment with retinoschisis spanning the entire macula. No signs of PVD were noted. Eleven months later, the foveal detachment has almost resolved and both the inner and outer retinoschisis has resolved. Twenty months after the initial visit, the foveal detachment has completely resolved. A residual vitreoretinal adhesion (between two red arrows) left after partial PVD can be seen. Twenty-six months of follow-up, the retinoschisis and vitreoretinal adhesion (between two red arrows) are stable. **Patient 3:** Resolution of a macular retinoschisis in an eye with a disruption of the epiretinal membrane (ERM). Left eye with an axial length of 31.46 mm in a man (51-year-old). OCT scan in both the vertical and horizontal sections at baseline showed a shallow retinoschisis with tractional ERM. Eleven months later, disruptions are noted in the ERM (red arrow) nasal and inferior to the fovea. The foveal retinoschisis has disappeared and the extrafoveal retinoschisis has been resolved.

Among the 120 eyes with MTM, resolution of MTM occurred in 5 patients (4.17%) with a mean duration of 19.8 months (range, 6–46 months). The mean age was 54.60 ± 3.91 years ranging from 49 to 70 years. The average SE was −16.10 ± 2.12 D ranging from −23.50 to −11.50 D and the average axial length was 30.63 ± 1.12 mm with a range from 27.05 to 32.86 mm. The OCT changes in these eyes included a decrease in the height of the macular retinoschisis in 2 eyes, a complete resolution of the macular retinoschisis in 2 eyes, and a complete resolution of foveal detachment in one eye. Two eyes were accompanied with ILM detachment and experienced spontaneous disruption of the detached ILM during the follow-up period, two eyes were accompanied with ERM and experienced spontaneous disruption of ERM during the follow-up period, and the remaining one eye developed partial posterior vitreous detachment (PVD) during the follow-up period. Representative cases are shown in Figure 4. For retinoschisis grading, resolutions were found in none of the S0 eyes, 3.33% of the S1 eyes, none of the S2 eyes, 13.64% of the S3 eyes, and 4.00% of the S4 eyes. The differences in the improvement rate among the 5 groups were not significant (*p* = 0.15, Fisher's exact test). In terms of foveal complications, eyes with foveal detachment showed the highest resolution rate, whereas eyes with isolated retinoschisis and lamellar MH showed a lower resolution rate (16.67, 3.61, and 0%; *p* = 0.1).

The changes of OCT features in patients with different prognosis during the follow-up period are shown in Table 3. The eyes with MTM resolution during the follow-up period experienced spontaneous ILM disruption at a significantly higher rate than the other two groups (*p* = 0.001, Fisher's exact probability test). Similarly, a significantly higher incidence of spontaneous ERM disruption was observed in the eyes with MTM resolution compared with the other two groups (*p* = 0.03, Fisher's exact probability test). There was no significant difference in the rate of PVD development among the groups that showed improvement, no change, or progression during the course. One eye experienced MTM progression, despite complete PVD development during the follow-up period.

**Table 3:** Changes of OCT features in patients with different prognosis.

**OCT Changes during the Follow-up Period**	**Prognosis**			
	**Progressed (32 eyes)**	**Stable (83 eyes)**	**Improved (5 eyes)**	***P-*value**
Spontaneous disruption of ILM	0 (0%)	0 (0%)	2 (40.00%)	**0.001** ^*^
ILM detachment	2 (6.25%)	0 (0%)	0 (0%)	>0.05
Spontaneous disruption of ERM	1 (3.13%)	4 (4.82%)	2 (40.00%)	**0.03** ^*^
Posterior vitreous detachment	1 (3.13%)	1 (1.20%)	1 (20.00%)	>0.05
Vitreomacular traction	1 (3.13%)	0 (0%)	0 (0%)	>0.05

*OCT, optical coherence tomography; LMH, lamellar macular hole; ILM, internal limiting membrane; ERM, epiretinal membrane*.

* *Fisher's exact tests*.

*Statistically significant p-values were reported in bold*.

## Discussion

By comprehensively evaluating the state of the vitreous, retina, and fovea on OCT, we found that ILM detachment and retinoschisis involved the entire macula at baseline were predictors for MTM progression. On the other hand, a higher proportion of spontaneous ILM disruption were found in the eyes with MTM resolution. These suggest that ILM may play a crucial role as a biomarker in the evolution of MTM. In addition, patients with foveal detachment at baseline were found to have a higher possibility of progression at a rapid speed, and a higher possibility of resolution as well, indicating that this was an unstable stage and required close follow-up. To the best of our knowledge, we first demonstrate the significance of ILM in the evolution of MTM. We believe that this study will contribute to a better understanding of the pathogenesis of MTM and provide references for predicting prognosis and surgical decision-making of MTM.

To date, there are few studies that have documented the progression of MTM using longitudinal data and the reported progression rate was quite different, ranging from 11.6 to 69.0% (9–11). Gaucher et al. (9) conducted a long-term follow-up of 50 eyes of 38 patients with myopic retinoschisis, among whom 14 eyes (28%) experienced progression in terms of visual acuity at the end of the follow-up. Shimada et al. (10) followed 207 eyes with MTM and found that the progression rate was only 11.6% during the follow-up. In this study, retinoschisis was divided into five grades (S0–S4) according to the location and extent of retinoschisis. Among the patients, 42.5% of them were not fovea involved (milder retinoschisis) and only 13.5% of them were entirely macula involved. Parolini et al. (11) studied the evolution of MTM in 72 eyes and found that the retinal pattern evolved from inner/outer maculoschisis to predominantly outer maculoschisis, then maculoschisis-macular detachment, and finally macular detachment. In this study, we followed 120 patients with MTM and found that the progression rate was 26.67%, which was different from previous studies. The reason may be related to the sample size and the composition of cases. The prognosis of MTM was affected by various factors. Among them, the vitreoretinal interface factors, which represented inner ocular force, are one of the most important independent factors. However, the pattern of vitreoretinal interface, including VMT, ERM and ILM detachment, was less evaluated in previous studies.

In this study, we comprehensively evaluated the pattern of the vitreoretinal interface, retina, and fovea on OCT and found that ILM detachment was one of the predictors for MTM progression. The ILM acts as a basal membrane for retinal Müller cells. Its major components are collagen types IV, VI, and VIII, associated with glycoproteins. Proper ILM stiffness is vital for the mechanical balance and stability between the vitreoretinal interface, while rigid ILM may play an important role in vitreoretinal abnormalities such as MTM. Our previous studies found that in eyes with MTM, the ILM presented abnormal ultrastructure including long irregular indentations and increased cell debris, which might result from Müller cell reactive gliosis responding to mechanical stress during the elongation of axial length in pathologic myopia (12). In the meantime, we found that removal of ILM during pars plans vitrectomy is beneficial to the recovery of postoperative anatomy and visual function for patients with MTM (13). These suggested that ILM is vital in the pathogenesis of MTM. In this study, we found that ILM detachment is a risk factor for MTM progression; meanwhile, MTM resolution can occur after spontaneous ILM disruption. These findings further verified that pathological ILM is not only involved in the occurrence of MTM, but also a risk factor for MTM development. We proposed that pathological ILMs with increased stiffness generate a centripetal traction in retina, contributing to the retinal splitting in pathologic myopia. In contrary, released traction resulting from spontaneous disruption of ILM that could lead to MTM resolution. Thus, monitoring the state of the ILM might help us to better predict the prognosis and make surgical decision for MTM.

Foveal detachment is a severe complication of pathologic myopia, which is often accompanied by serious visual impairment and poor prognoses (14). Huang et al. (15) found that preoperative foveal detachment is one of the risk factors for MH retinal detachment after vitrectomy in MTM. This study found that patients with foveal detachment were more likely to experience progression at a rapid speed (5/12, 41.67%). Similarly, Shimada et al. (10) studied 10 cases of foveal detachment and found a progression rate of 100% during a mean follow-up duration of 31.7 months. Interestingly, we found that this group of patients was more likely to experience resolution (16.67%) as well. No significant difference was detected in age and in axial length between the eyes with and without foveal detachment. This was consistent to Lai et al. (16), who reported eight cases of foveal detachment experienced spontaneous resolution during the follow-up. These revealed that foveal detachment is an unstable intermediate stage in the development of MTM. In a mechanical view, in eyes with foveal detachment, the outer retina exhibited a steep, curved shape with an undulated surface. We proposed that the sharp changes of curvature in the outer retina at the detached region may contribute to the lower structural stability of foveal detachment. Thus, this group of patients needs intensive follow-up and timely intervention.

Resolution of MTM is a relatively rare phenomenon. Previous studies were mostly based on case reports and only one study (11) reported the incidence of spontaneous resolution of MTM in a large sample. The reported resolution rate of 3.9% was similar with the rate of 4.2% obtained in this study. In 2003, the resolution of MTM without surgical intervention was first reported by Polito et al. (17). They reported a spontaneous resolution after PVD in a patient with foveoschisis and foveal detachment. Subsequently, spontaneous resolution in cases with foveoschisis, foveal detachment, and even MH retinal detachment was reported (16, 18). At present, it is believed that multiple factors result in the resolution of MTM including the release of anterior traction and the morphological changes of posterior sclera (18). The 5 cases with resolution observed in this study all experienced the release of anterior traction such as the disruption of the anterior ERM, the disruption of the detached ILM, and partial PVD. However, little is known when a spontaneous resolution will develop and further exploration with larger sample size is needed.

This study has several limitations. Given the retrospective design of this study, durations of follow-up were variable. Further prospective investigations with larger sample size would be demanded.

In summary, this study found that during a mean follow-up period of 15.4 months, the rate of progression and resolution in MTM were 26.67 and 4.17%, respectively. The ILM detachment is a risk factor for the MTM progression, whereas the MTM resolution can occur after the spontaneous disruption of ILM, indicating that ILM might be one of the biomarkers of the evolution of MTM. Patients with foveal detachment are unstable and require close follow-up and timely intervention. By comprehensively analyzing the OCT-based structural alterations, namely, the retinoschisis grading, the foveal complications and vitreoretinal interface abnormalities, and investigating the risk factors for MTM progression, we believe that this study will contribute to a better understanding of the pathogenesis of MTM and provide references for predicting prognosis and surgical decision-making of MTM.

## Data Availability Statement

The original contributions presented in the study are included in the article/supplementary material, further inquiries can be directed to the corresponding author/s.

## Ethics Statement

The studies involving human participants were reviewed and approved by the Institutional Review Board of Shenzhen Eye Hospital (Shenzhen, China). Written informed consent for participation was not required for this study in accordance with the national legislation and the institutional requirements.

## Author Contributions

DF contributed to the study design and wrote the manuscript. JS contributed to the clinical data collection, data analyses, and manuscript polishing. LC contributed to manuscript preparation and clinical data collection. SZ contributed to the conception of the study, study design, and manuscript polishing. All authors have read and approved the final version of the manuscript.

## Funding

This study was supported by grants from the Sanming Project of Medicine in Shenzhen (SZSM202011015), the Natural Science Foundation of Guangdong Province (2021A1515011090), and the National Natural Science Foundation of China (81900877). These funding organizations had no role in the design or conduct of this study.

## Conflict of Interest

The authors declare that the research was conducted in the absence of any commercial or financial relationships that could be construed as a potential conflict of interest.

## Publisher's Note

All claims expressed in this article are solely those of the authors and do not necessarily represent those of their affiliated organizations, or those of the publisher, the editors and the reviewers. Any product that may be evaluated in this article, or claim that may be made by its manufacturer, is not guaranteed or endorsed by the publisher.
